# PCV13, PCV15 or PCV20: Which vaccine is best for children in terms of immunogenicity?

**DOI:** 10.14745/ccdr.v50i12a04

**Published:** 2024-01-01

**Authors:** Philippe De Wals

**Affiliations:** 1 Department of Social and Preventive Medicine, Laval University, Québec City, QC; 2 Institut national de Santé publique du Québec, Québec City, QC; 3 Centre de recherche du Centre hospitalier universitaire de Sherbrooke, Sherbrooke, QC

**Keywords:** **Keywords:** pneumococcal conjugate vaccine, immunogenicity, randomized trial, opsonophagocytic activity

## Abstract

**Background:**

The new 15- and 20-valent pneumococcal conjugate vaccines (PCV15 and PCV20) have been marketed on the basis of immunogenicity criteria, one of them being a non-inferior response as compared with the 13-valent vaccine (PCV13). In the past, PCV13 was also authorized on the basis of the same criteria, using the 7-valent vaccine (PCV7) as a reference.

**Methods:**

Our aim was to compare the immunogenicity of these three vaccines in toddlers. Functional opsonophagocytic activity (OPA) titre ratios measured in the same and different randomized trials were computed to assess the respective immunogenicity of these four products.

**Results:**

Results suggest that both PCV15 and PCV20 are less immunogenic than PCV13 for most common serotypes and that the two new vaccines induce a broadly similar response. The PCV7 vaccine was already slightly more immunogenic than PCV13 meaning that PCV15 and PCV20 compare poorly with PCV7. Results also point towards a reduced immunogenicity of the 2+1 dose schedule compared to the 3+1 dose schedule for PCV13, PCV15 and PCV20.

**Conclusion:**

Post-marketing studies will have to be conducted to assess the effectiveness of PCV15 and PCV20 and their real-life benefit over PCV13.

## Introduction

The first pneumococcal conjugate vaccine containing seven serotypes (PCV7) was authorized in 2000, according to a 3+1 dose schedule in infants. The authorization was based on a Phase 3 randomized clinical trial (RCT) demonstrating a protective efficacy of 97.4% (95% CI: 82.7%–99.9%) against invasive pneumococcal disease caused by vaccine serotypes in the intent-to-treat analysis ((1)). For ethical and feasibility reasons, the 13-valent vaccine (PCV13) containing the same carrier protein as PCV7 (CRM_197_) was authorized in 2010 on the basis of immunogenicity criteria rather than the demonstration of clinical protection ((2)). In 2005, a first set of immunogenicity criteria was proposed by the World Health Organization (WHO) for the licensure of new pneumococcal conjugate vaccines and used for marketing the new 15-valent (PCV15) and 20-valent (PCV20) conjugate vaccines in 2022–2023 ((3)). One of these criteria is the demonstration of a non-inferior immune response when compared to a registered vaccine. The non-inferiority requirement applies to serotype-specific proportions of responders, Immunoglobulin G (IgG), and functional antibody levels. For antibody levels, non-inferiority is declared if the lower limit of the two-sided 95% CI of the new/old geometric mean ratio is above 0.5 (3). However, non-inferiority does not mean equivalence, and the sequential use of PCV7, followed by PCV13, as references for the authorization of newer vaccines may have cumulative negative consequences on the level of protection and its duration. In this commentary, functional opsonophagocytic activity (OPA) titre ratios measured in RCTs pertaining to PCV7, PCV13, PCV15 and PCV20 were compared to assess the respective performance of these four products in terms of immunogenicity, which is associated with clinical effectiveness.

## Analysis

The comparison of mean OPA titres one month after the toddler dose in three PCV trials using a 3+1 dose schedule (2, 4, 6 and 12–15 months) is presented in **Table 1**. The first comparison comes from the pivotal United States (US) study on the safety, tolerability, and immunogenicity of PCV13 with PCV7 as a reference, the two vaccines having been administered with routine paediatric vaccinations, according to the US-recommended infant vaccination schedule at that time (4). Post-booster means OPA titres were lower with PCV13 than with PCV7 for six of the seven common serotypes (19F was the exception), with an average PCV13/PCV7 OPA ratio of 0.77. The second comparison comes from a Phase 3, multicentre trial aiming to evaluate the safety, tolerability, and immunogenicity of a four-dose regimen of PCV15 using PCV13 as a comparator ((5)). With the exception of serotype 14, all PCV15/PCV13 OPA ratios were below one, with an average value of 0.75. The third trial was a Phase 2 study on the safety and immunogenicity of PCV20 using PCV13 as a comparator in healthy infants in the US ((6)). Overall, OPA titres with PCV20 were lower than those observed with PCV13, with an average PCV20/PCV13 ratio of 0.72 for the common serotypes. Using the results of the two latest trials, it is possible to compare PCV15 with PCV20 for the 13 serotypes included in PCV13. As seen in Table 1, most PCV15/PCV20 ratios were close to one, with the exception of serotype 14 (ratio=1.82). The average PCV15/PCV20 ratio was 1.04, suggesting that the two new vaccines have rather similar immunogenicity. When their immunogenicity was compared to that of PCV7 for the seven common antigens, however, a reduced immunogenicity was observed, with a mean PCV15/PCV7 ratio of 0.63 and a mean PCV20/PCV7 ratio of 0.54.

**Table 1:** Comparison of mean geometric opsonophagocytic activity, titres one month after the toddler dose in trials using a 3+1 doses schedule (2, 4, 6 and 12–15 months)

Reference	Yeh *et al*., 2010			Lupinacci *et al*., 2023			Senders *et al.*, 2021			Indirect comparisons		
Serotype	OPA PCV13	OPA PCV7	Ratio PCV13/PCV7	OPA PCV15	OPA PCV13	Ratio PCV15/PCV13	OPA PCV20	OPA PCV13	Ratio PCV20/PCV13	Ratio PCV15/PCV20	Ratio PCV15/PCV7	Ratio PCV20/PCV7
	A	B	C=A/B	D	E	F=D/E	G	H	I=G/H	J=F/I	K=FxC	L=IxC
1	N/A	N/A	N/A	138.5	228.6	0.61	50.4	92.9	0.54	1.12	N/A	N/A
3	N/A	N/A	N/A	389.1	455.9	0.85	93.0	109.3	0.85	1.00	N/A	N/A
4	1,180	1,492	0.79	2,558.3	3,492.6	0.73	490.3	662.5	0.74	0.99	0.58	0.59
5	N/A	N/A	N/A	1,062.9	1,538.8	0.69	78.7	112.8	0.70	0.99	N/A	N/A
6A	N/A	N/A	N/A	5,553.5	7,784.6	0.71	1,671.4	2,155.8	0.78	0.92	N/A	N/A
6B	3,100	4,066	0.76	4,641.8	5,897.0	0.79	1,354.9	1,808.1	0.75	1.05	0.60	0.57
7F	N/A	N/A	N/A	10,098.6	12,301.9	0.82	2,590.7	3,280.7	0.79	1.04	N/A	N/A
9V	11,856	18,032	0.66	1,714.5	4,237.1	0.40	1,280.2	2,030.0	0.63	0.64	0.27	0.41
14	2,002	2,366	0.85	4,558.1	3,010.5	1.51	938.8	1,127.9	0.83	1.82	1.28	0.70
18C	993	1,722	0.58	2,471.0	3,319.6	0.74	2,016.2	2,703.3	0.75	1.00	0.43	0.43
19A	N/A	N/A	N/A	3,370.4	5,584.6	0.60	651.3	874.8	0.74	0.81	N/A	N/A
19F	200	167	1.20	2,286.4	2,626.7	0.87	500.5	751.0	0.67	1.31	1.04	0.80
23F	2,723	4,982	0.55	6,098.6	13,677.9	0.45	693.1	1,253.9	0.55	0.81	0.24	0.30
**Mean of ratios**	N/A	N/A	**0.77**	N/A	N/A	**0.75**	N/A	N/A	**0.72**	**1.04**	**0.63**	**0.54**
**Median of ratios**	N/A	N/A	**0.76**	N/A	N/A	**0.73**	N/A	N/A	**0.74**	**1.00**	**0.58**	**0.57**

Abbreviations: N/A, not applicable; OPA, opsonophagocytic activity; PCVY, 7-valent vaccine; PCV13, 13-valent vaccine; PCV15, 15-valent vaccine; PCV20, 20-valent vaccine

The 2+1 PCV13 schedule was authorized on the basis of a comparison with the 3+1 PCV13 schedule. A direct comparison between PCV13 and PCV7 for this schedule is not available. As seen in **Table 2**, both PCV15 and PCV20 generated lower OPA titres than PCV13 for a majority of the common serotypes in the two pivotal Phase 3 trials supporting their respective authorization in a 2+1 schedule ((7,8)). The mean PCV15/PCV13 ratio was 0.75, similar to the mean PCV20/PCV15 ratio of 0.76.

**Table 2:** Comparison of mean geometric opsonopgagocytic activity, titres one month after the toddler dose in trials using a 2+1 doses schedule (2, 4, and 11–15 months)

Reference	Martinon-Torres *et al.*, 2023			Pfizer, NCT04546425 results, 2023			Ratio PCV15/PCV20
Serotype	OPA PCV15	OPA PCV13	Ratio PCV15/PCV13	OPA PCV20	OPA PCV13	Ratio PCV20/PCV13	
	A	B	C=A/B	D	E	F=D/A	G=C/F
1	136.8	164.6	0.83	54	101	0.53	1.55
3	321.5	303.0	1.06	99	129	0.77	1.38
4	2,231.7	3,206.4	0.70	904	992	0.91	0.76
5	791.6	947.9	0.84	60	82	0.73	1.14
6A	3,274.9	5,387.2	0.61	1,101	1,304	0.84	0.72
6B	2,439.9	3,182.4	0.77	537	864	0.62	1.23
7F	6,300.9	10,071.7	0.63	1,811	2,197	0.82	0.76
9V	1,904.4	2,616.6	0.73	3,254	4,544	0.72	1.02
14	2,638.8	2,682.1	0.98	738	920	0.80	1.23
18C	1,968.6	2,091.8	0.94	1,296	1,870	0.69	1.36
19A	2,995.6	4,254.3	0.70	754	707	1.07	0.66
19F	1,793.9	4,254.3	0.42	183	258	0.71	0.59
23F	4,517.8	7,987.6	0.57	697	975	0.71	0.79
**Mean of ratios**	N/A	N/A	**0.75**	N/A	N/A	**0.76**	**1.02**
**Median of ratios**	N/A	N/A	**0.73**	N/A	N/A	**0.73**	**1.02**

Abbreviations: N/A, not applicable; OPA, opsonophagocytic activity; PCV13, 13-valent vaccine; PCV15, 15-valent vaccine; PCV20, 20-valent vaccine

From results presented in Table 1 and Table 2, it is possible to compare the immunogenicity of the 3+1 and 2+1 schedules. For PCV13, the average 3+1/2+1 OPA ratio was 1.39 in the two trials conducted by Merck ((5,7)), and the mean ratio was 1.35 in the two trials conducted by Pfizer ((6,8)). For PCV15, the average 3+1/2+1 OPA ratio was 1.35 in the two trials conducted by Merck ((5,7)). For PCV20, the average 3+1/2+1 OPA ratio was 1.31 in the two trials conducted by Pfizer ((6,8)). These results point towards a reduced immunogenicity of the 2+1 dose schedule compared to the 3+1 dose schedule following the toddler booster dose.

## Discussion

Studies based on a face-to-face comparison of the two new PCV15 and PCV20 in infants are unavailable. Results presented here from indirect comparison with PCV13 as a common reference do suggest that both PCV15 and PCV20 are less immunogenic than PCV13 for most common serotypes and that the two new vaccines induce a broadly similar response. The first PCV7 conjugate product was already slightly more immunogenic than PCV13 for their common antigen, meaning that PCV15 and PCV20 compare poorly with PCV7. Several biological mechanisms have been proposed to explain the negative interference resulting from an increase in the number of bacterial polysaccharides included in conjugate vaccines, including a “carrier-induced-epitopic suppression” that may occur when the response to the polysaccharide is diminished in a competition with the anti-peptide-carrier response, a problem that may be aggravated by prior exposure to the carrier from another vaccination ((9,10)). A reduced immune response may negatively affect the short-term protection provided by a particular vaccine schedule, the duration of protection, and the herd immunity at population levels, especially for pneumococcal serotypes that are less sensitive to vaccine-induced antibodies such as ST3, ST7F, ST19A and ST19F ((11)). Following a recent National Advisory Committee on Immunization (NACI) statement published in March 2023, discussions are underway in all Canadian jurisdictions as to which PCV to select for children ((12)). Besides economic considerations and serotype coverage that will certainly be dominant arguments in the vaccine selection, the strength of the immunologic response must also be looked at, although the exact clinical meaning of observed differences is difficult to predict.

Another interesting observation is the lower immunogenicity of the 2+1 immunization schedule compared with the 3+1 schedule, as shown for PCV13, PCV15 and PCV20. In a case-control study performed during a period of shortage of PCV7 in the US, many children received less than the recommended four doses. There was a minimal difference in the effectiveness of two doses given before eight months of age with a booster dose given at 12–16 months (98%; 95% CI: 75%–100%) and three doses given before eight months of age with a booster dose given at 12–16 months (100%; 95% CI: 94%–100%) ((13)). For economic considerations and to decrease the total number of vaccines administered to children, a 2+1 PCV schedule is now accepted as a standard of care for healthy children by the WHO (14)). In January 2020, a 1+1 immunization PCV13 schedule (3 and 12 months of age) was introduced in the United Kingdom, replacing the 2+1 schedule, on the basis of an immunogenicity trial and the effect on nasopharyngeal carriage ((15)). The effectiveness of this reduced schedule remains to be seen.

The approach selected here to compare immune responses in different trials have also been used in a recently published paper comparing OPA responses following one PCV15 dose or one PCV20 dose in adults, although a more sophisticated statistical analysis was performed in the comparison ((16)). The OPA measurement is recognized as a better predictor of the clinical effectiveness of PCVs than anti-capsular polysaccharide antibody levels determined by the enzyme-linked immunosorbent assay (ELISA), although the latter method has the advantage of being standardized for inter-laboratory comparisons ((17,18)). Mean geometric OPA titres cannot be compared between different laboratories and between different serotypes in a same laboratory. However, the use of serotype-specific ratios of titres generated by different vaccines measured in a same laboratory, at the same time, overcomes these difficulties.

One limitation of this short commentary is that confidence intervals of ratios are not presented. In Phases 2/3 immunogenicity trials aiming to demonstrate a non-inferior immune response of an investigational vaccine compared to a registered product, several hundred participants are typically recruited. The number of participants in each arm of the trials reported in Table 1 and Table 2 ranged from a minimum of 230 to a maximum of 860 ((5,6)). The calculation of ratios of means from independent samples generates much larger confidence intervals than those obtained for mean estimates in each of the samples, and this problem is even more important when ratios of ratios are computed ((19)). Also, multiple comparisons as reported in Table 1 (n=60) and in Table 2 (n=26), mean that more stringent *p*-values would have to be applied to declare a statistically significant result, less than 0.0008 and 0.002, respectively, with the Bonferroni correction ((20)). The interpretation of results in this analysis has thus to therefore been made on trends rather than on individual estimates.

## Conclusion

Results suggest that both PCV15 and PCV20 are less immunogenic than PCV13 and especially PCV7 for most common serotypes, and that the two new vaccines induce a broadly similar response. The increasing number of pneumococcal polysaccharides included in conjugate vaccines is associated with a trend towards reduced immunogenicity. Means to circumvent this problem include an increase in the polysaccharide dose, as made for an investigational 21-valent CRM_197_ PCV targeting serotypes found in adults ((21)), or the use of another protein-carrier and novel conjugation technique, as made for another investigational 24-valent pneumococcal vaccine ((22)). Several years will be needed before a possible marketing of extended newer-generation PCVs for children. In the meantime, Phase 4 post-marketing studies will have to be conducted to assess the effectiveness of PCV15 and PCV20 and their real-life benefit over PCV13.
